# Supplementary material for: Viral Population Diversity during Co-Infection of Foot-And-Mouth Disease Virus Serotypes SAT1 and SAT2 in African Buffalo in Kenya
Source: Viruses. 2022 Apr 25;14(5):897. doi: 10.3390/v14050897 (PMC9145140; doi:10.3390/v14050897)

Table S1. African buffalo metadata. Rows in bold refer to animals found to be co-infected with SAT1 and SAT2 FMDV viruses via qRT-PCR and deep sequencing. The age of the animals are in years.

Animal ID	Date Collected	Age	Sex	Buffalo herd ID	SAT Serotype	Sequence name
5	1/8/2016	3.5	F	LODRU3	1	5/Kenya/8Jan2016/SAT1
**6**		**2.5**	**F**		**1 and 2**	**6/Kenya/8Jan2016/SAT1 6/Kenya/8Jan2016/SAT2**
7		2	F		1	7/Kenya/8Jan2016/SAT1
8		1.5	M		1	8/Kenya/8Jan2016/SAT1
10		4	F		1	10/Kenya/8Jan2016/SAT1
12	1/10/2016	3	F	GRANTS4	2	12/Kenya/10Jan2016/SAT2
13		3	F		2	13/Kenya/10Jan2016/SAT2
14		0.7	M		2	14/Kenya/10Jan2016/SAT2
18		1.5	F		2	18/Kenya/10Jan2016/SAT2
19		2.5	M		2	19/Kenya/10Jan2016/SAT2
20		2.5	F		2	20/Kenya/10Jan2016/SAT2
23		2.5	M		2	23/Kenya/10Jan2016/SAT2
25	1/11/2016	3.5	F	LODRU5	1	25/Kenya/11Jan2016/SAT1
26		2	M		1	26/Kenya/11Jan2016/SAT1
27		5	F		1	27/Kenya/11Jan2016/SAT1
28		4	F		1	28/Kenya/11Jan2016/SAT1
31		7	M		1	31/Kenya/11Jan2016/SAT1
32		2	F		1	32/Kenya/11Jan2016/SAT1
34	1/12/2016	3.5	M	GRANTS4	2	34/Kenya/12Jan2016/SAT2
35		7	M		2	35/Kenya/12Jan2016/SAT2
**36**		**5**	**F**		**1 and 2**	**36/Kenya/12Jan2016/SAT1 36/Kenya/12Jan2016/SAT2**
37		6	F		1	37/Kenya/12Jan2016/SAT1
38		4	F		1	38/Kenya/12Jan2016/SAT1
41		1.5	F		2	41/Kenya/12Jan2016/SAT2
42		1.5	F		2	42/Kenya/12Jan2016/SAT2
43		7	M		2	43/Kenya/12Jan2016/SAT2
46		6	M		2	46/Kenya/12Jan2016/SAT2
48		5	F		1	48/Kenya/12Jan2016/SAT1
50		10	F		2	50/Kenya/12Jan2016/SAT2
**51**		**4**	**F**		**1 and 2**	**51/Kenya/12Jan2016/SAT1 51/Kenya/12Jan2016/SAT2**
54		4	M		2	54/Kenya/12Jan2016/SAT2
55		4	F		1	55/Kenya/12Jan2016/SAT1
56		4	F		2	56/Kenya/12Jan2016/SAT2
58		8	M		2	58/Kenya/12Jan2016/SAT2
**59**		**4.5**	**M**		**1 and 2**	**59/Kenya/12Jan2016/SAT1 59/Kenya/12Jan2016/SAT2**
60	1/13/2016	3.5	F	SCOTTS6	2	60/Kenya/13Jan2016/SAT2
**61**		**2**	**M**		**1 and 2**	**61/Kenya/13Jan2016/SAT1 61/Kenya/13Jan2016/SAT2**
62		3	M		2	62/Kenya/13Jan2016/SAT2
63		4.5	F		2	63/Kenya/13Jan2016/SAT2
65		2.5	F		2	65/Kenya/13Jan2016/SAT2
67	1/13/2016	6	M	MORANI7	2	67/Kenya/13Jan2016/SAT2
69		4.5	F		2	69/Kenya/13Jan2016/SAT2
71		2	F		2	71/Kenya/13Jan2016/SAT2
72		5	M		1	72/Kenya/13Jan2016/SAT1
73	1/13/2016	5	M	MORANI8	1	73/Kenya/13Jan2016/SAT1
74		1.5	F		2	74/Kenya/13Jan2016/SAT2
75		6	M		2	75/Kenya/13Jan2016/SAT2
76		2.5	M		2	76/Kenya/13Jan2016/SAT2
80	1/13/2016	3	M	ZEBRA PLAINS9	2	80/Kenya/13Jan2016/SAT2
81		3	F		2	81/Kenya/13Jan2016/SAT2
83		3	M		2	83/Kenya/13Jan2016/SAT2
86		3.5	M		2	86/Kenya/13Jan2016/SAT2
87		5	F		2	87/Kenya/13Jan2016/SAT2
88		2	F		2	88/Kenya/13Jan2016/SAT2
K14	1/10/2014	X	X	X	1	K14/Kenya/10Jan2014/SAT1
K29	1/29/2014	X	X	X	1	K29/Kenya/29Jan2014/SAT1

Figure S1. FMDV VP1 phylogenies with study sequences to determine SAT1 and SAT2 topotypes. Tips highlighted in red squares correspond to the sequences included in this study where A) indicates the SAT1 VP1 phylogeny and B) indicates the SAT2 VP1 phylogeny. Topotypes are designated by vertical blue lines and labeled accordingly.

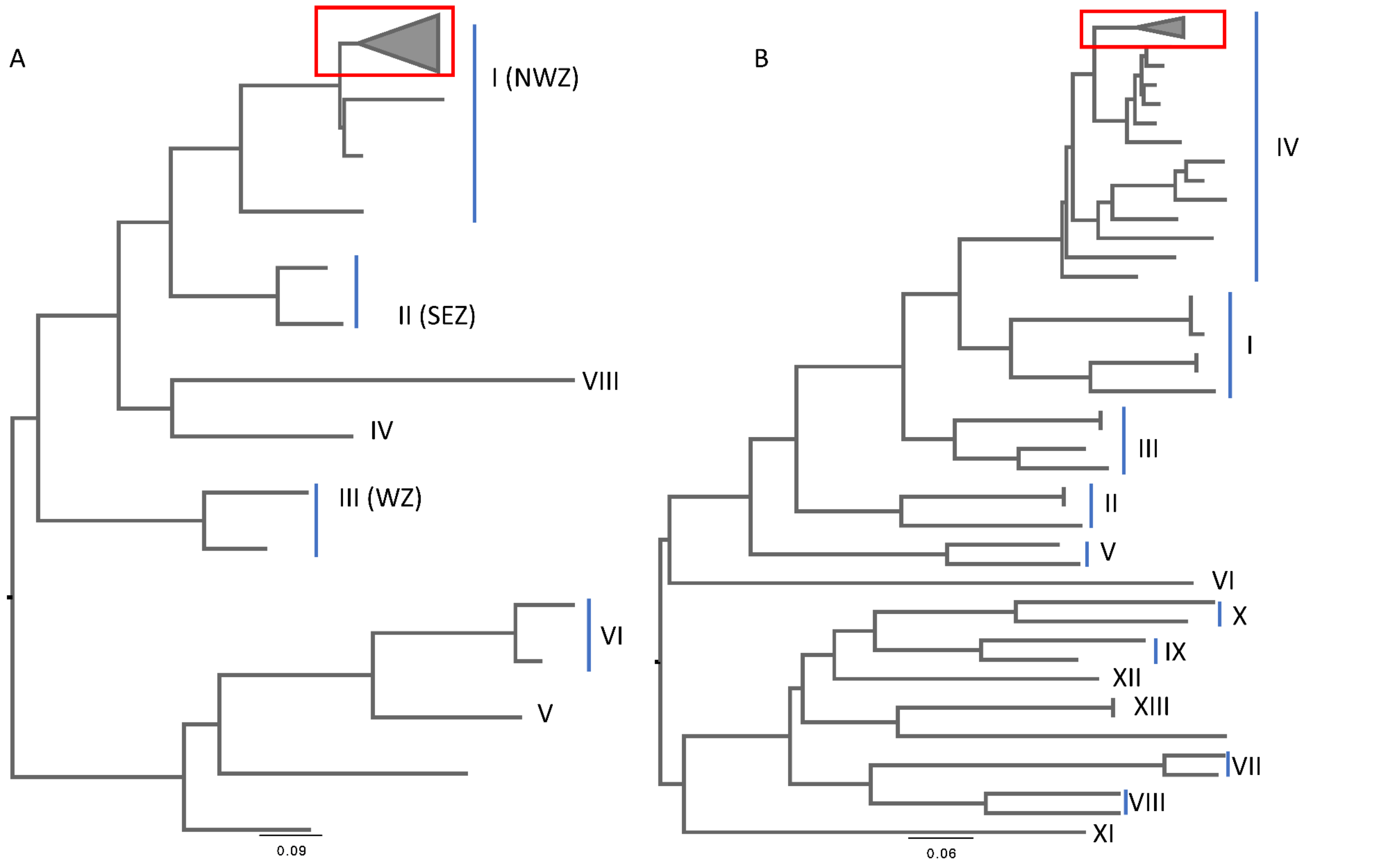

Figure S2. Maximum Likelihood Phylogenetic trees of FMDV protein coding regions. All phylogenetic trees were performed using the best-fit model as determined by Mega v10.0 using 500 bootstrap replicates. Branch support is displayed if above 70. For animals 36 and 59, groups of sequences within a serotype are designated by Group 1 (G1) or Group 2 (G2). The first passage sequences are indicated as 'passage.' Regions A) Lpro, B) VP1-VP4, C) 2B-2C, D) 3A-3B and E) 3C-3D were used for the analysis.

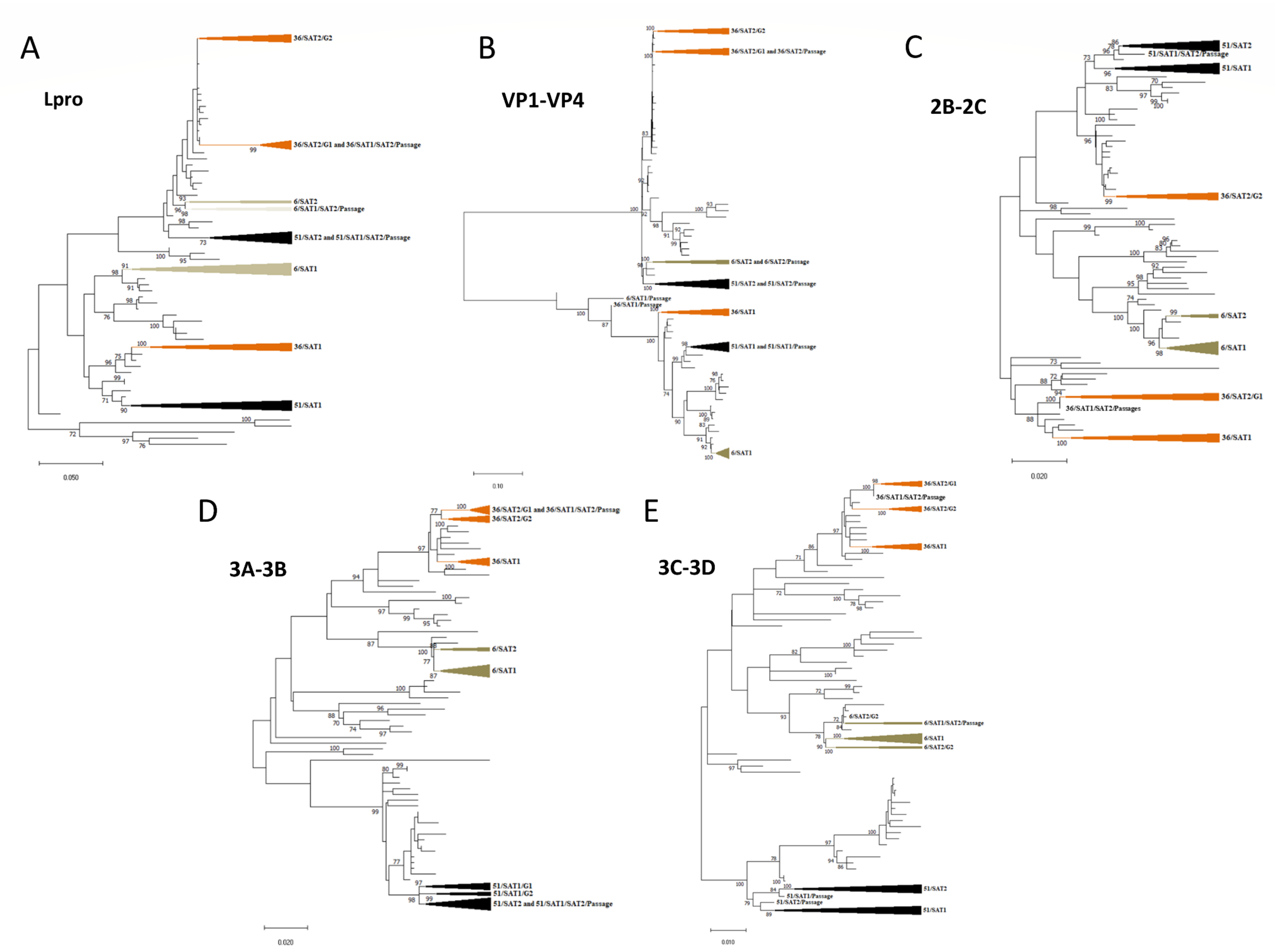

Figure S3. Recombination detection using Simplot. Recombination analyses were carried out using a 200bp window, 20bp step, a Kimura (2-parameter) model and T/t of 2.0. The position of the FMDV protein-coding regions are designated at the bottom of each graph with black or grey bars. The graphs queries are indicated above the graphs.

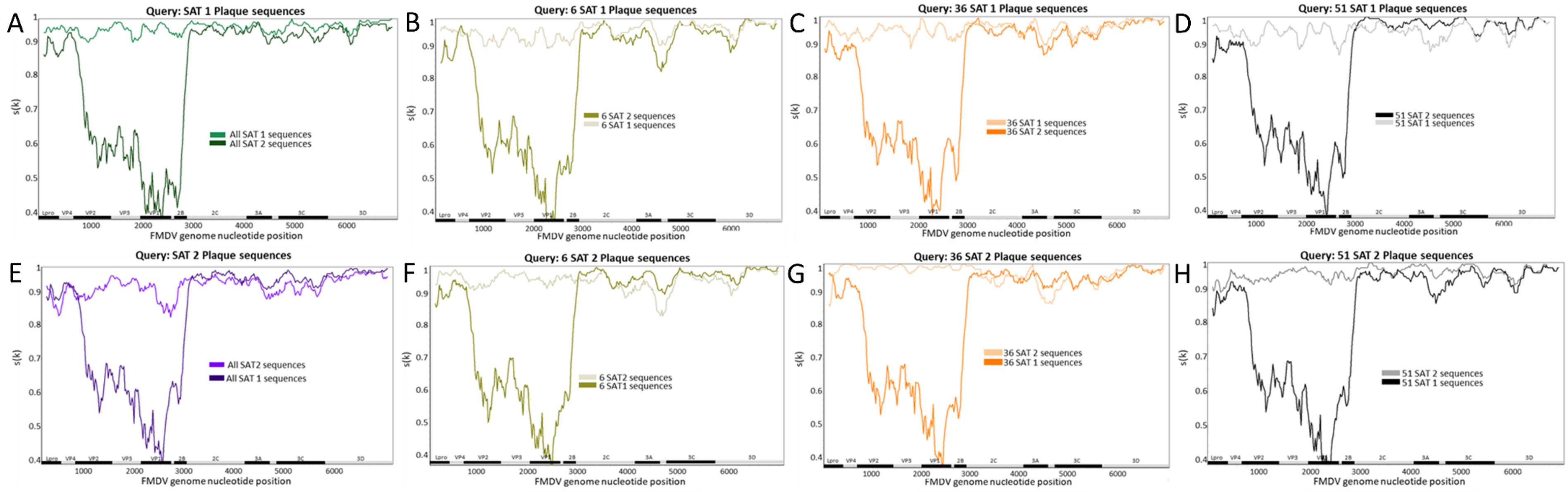

Supplement: Supplementary file 1 [file viruses-14-00897-s001.zip › viruses-1679069-supplementary.pdf]
